# Ten Simple Rules for Developing Public Biological Databases

**DOI:** 10.1371/journal.pcbi.1005128

**Published:** 2016-11-10

**Authors:** Mohamed Helmy, Alexander Crits-Christoph, Gary D. Bader

**Affiliations:** 1 The Donnelly Centre, University of Toronto, Toronto, ON, Canada; 2 Department of Biophysics, Johns Hopkins University, Baltimore, MD, United States of America

## Introduction

Biological databases are online libraries that contain structured information about living organisms. These databases are indispensable research tools, as they provide convenient, computable access to prior knowledge that is vital for planning future experiments and for discovering new knowledge through data mining—they help us “stand on the shoulders of giants.” Because of their importance to research, the number of public biological databases is increasing. For instance, the number of biological databases published per year in the journal *Nucleic Acid Research* (*NAR*) increased dramatically from only two databases in 1980 to 182 in 2016, with the expectation that this single journal will have published over 2,500 database articles by the end of 2017 [1]. Some of these databases are key, sophisticated, user-friendly, long-term, stable resources, built and maintained by professional teams. However, others have been criticized for being difficult to use or having unclear data quality levels [2,3], and many become obsolete over time [4]. So, if you are considering developing a new database, and especially if you are a student or postdoc, please, for the love of science, follow these ten simple rules for creating and maintaining biological databases (and also a similar set of great rules for scientific web resources [5]).

### Rule 1: Don’t reinvent the wheel

**Fig 1:**
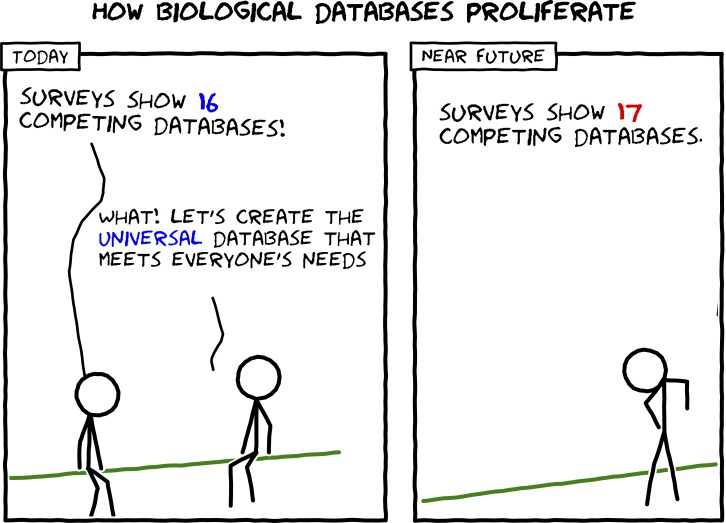
How biological databases proliferate (adapted from https://xkcd.com/927/ and drawn using Comix I/O [http://cmx.io/]).

Creating a high-quality database is a responsibility that involves strong commitment to accurate data collection and regular content and feature updates, not to mention a substantial time investment. The strongest reason to create a new database is scientific demand for a type of data not easily available in a computable form anywhere else. It is most useful to have all data of a single type in one easy-to-search location, so, ideally, everyone interested in collecting data about a specific topic should collaborate to create one resource, or at least should coordinate efforts to reduce duplication of work (Fig 1). Either way, the data content and software features you create will have the greatest impact if they are original and useful; thus, a comprehensive literature review is a necessary starting point that comes before any actual work (as in any scientific endeavor). A good place to start searching for relevant prior work is the “NAR Online Molecular Biology Database Collection,” which, as of January 2016, contains 1,666 biological databases organized into categories [1], collected from the annual *NAR* database issue. *NAR* also publishes an annual web server issue dedicated to web-based software resources [6], and the journal *Database* focuses on biological databases and curation. Several online directories maintain biological database link collections, such as Pathguide (547 databases) [7], The Tools and Data Service Registry (557 databases) [8], The Bioinformatics Links Directory (623 databases) [9], *OMICtools* (1,513 databases) [10], and *MetaBase* (1,802 databases) [11].

### Rule 2: The three most important things in database development are data quality, data quality, and data quality

Some databases collect unique content directly from experiments via authors (e.g., GenBank [12]) or expertly curated from the literature (here, we call these “primary”), and some collect non-unique content from other databases (we call these “secondary” or meta-databases). Primary data could be collected from a community of data generators, such as with GenBank [12], or curated by experts, such as with UniProtKB/Swiss-Prot [13]. Secondary databases, such as InterPro [14], host data collected from other public resources that are often formatted and processed in a uniform way, which can create a more comprehensive and useful data source. Depending on the database type, there are different standards for quality control. Primary databases are responsible for thoroughly checking the quality of all input data, for instance using manual cross-checking and rule-based automated data entry validation [15,16]. It is a good idea to develop a manual describing standard operating procedures for curation to help maintain and communicate quality standards. Secondary databases must ensure to not introduce new errors via their data integration process. Ideally, the integration process will improve data quality via cleaning and normalizing (e.g., standard database identifier use). In either case, following best practices and standards can help improve data quality. A data model standard may require that particular information (e.g., database or gene identifier, an ontology) is used, otherwise intended uses will not be possible. Often, an automatic data validator is available for a standard format to ensure that rules are followed, leading to more consistency and fewer errors. For meta-databases, transforming all incoming data to a standard format eases data integration and can reduce software development time and implementation errors, as well-tested software libraries are available to help read, write, and access the data. For example, the Proteomics Standards Initiative Molecular Interaction (PSI-MI) format captures molecular interactions, such as protein–protein interactions, and maintains a validator, a software library, and other tools to ease working with this data type [17]. Data provenance, information about where the data has come from and when it was updated, is also important to capture and track in order to help users critically evaluate database content quality.

### Rule 3: Know your audience

OK, so there is more to database development than data quality. Defining a target audience is critical for defining database scope and useful data access interfaces (e.g., web interface, application programming interface [API]). Use cases supporting this audience should then be defined, which can then be addressed clearly by what data types will be collected and what queries will be emphasized. Users of biological databases are often of three types: (a) biologists and clinical researchers interested in manual queries via the web, (b) computational biologists interested in batch data download and query systems, and (c) software developers interested in APIs. One study showed that two thirds of users of online biological resources have limited programming experience [5]. These users generally require a web interface that facilitates data access and retrieval. On the other hand, computational biologists and software developers generally need multiple channels for accessing data programmatically or in batch for use and integration into their own analysis workflows and software tools. For instance, NCBI provides the Entrez web interface for manual queries across many databases, a download site for batch data access, and an extensive web service API for programmatic access [18].

### Rule 4: Use modern technology

Most databases are available via an interactive user interface on the web catering to the majority of users. Modern users demand that websites adhere to very high technical and design standards, as is commonplace with websites like Facebook and Google. Websites should have intuitive graphical design and support standard web browsers and operating systems. They also must have a smooth and responsive graphical user interface (GUI) and be secure and robust. Popular technologies such as HTML5, CSS3, and JavaScript now make it straightforward to create such websites. Use of standard front-end technologies often automatically provides support for multiple browsers and operating systems and makes it easy to implement the concept of Responsive Web Design (RWD), which enables a website to adapt its layout to optimally fit different viewing environments, such as desktop, mobile browsers, or touch devices [19]. It is also now possible to build such websites quickly from reusable components. Front-end templates, such as Twitter Bootstrap, help create standardized pages, icons, symbols, graphical components, and fonts. JavaScript libraries (e.g., JQuery) help create GUI components, such as forms and animations, without much effort. Web application frameworks, such as Shiny for R and Django for Python, are collections of ready-made packages and tools that enable developers to automatically generate whole websites, thus greatly speeding up the development process and reducing the need for debugging and testing. Furthermore, several modern technologies, such as node.js, enable writing both the client and server side in one language, JavaScript, and come with thousands of packages providing powerful features.

There has also been a recent diversification in freely available database technologies. If your data content is naturally tabular with a few fields connecting each table with another, it is likely to be relational and can be stored in databases such as MySQL. If content is more naturally organized as free-text or structured documents, networks, key-value pairs, or other non-tabular data, “NoSQL” database technology may be a better choice. Some database systems also make it easy to replicate a database across multiple servers, making indexing and retrieval of large quantities of data faster, because operations can be parallelized. For instance, MongoDB stores structured documents and Apache Lucene indexes free text documents. Using modern technologies in interface and database development can create a speedier user experience and ease the technical maintenance of the system (see Rule 9).

### Rule 5: Put yourself in your user’s shoes

A common complaint of researchers using biological databases is that the interfaces to these resources are often difficult to navigate [2,3,20]. Databases with a good user experience make it easy to navigate and find data even by non-experts. Database navigation is facilitated by a graphical user interface that organizes, presents, and visualizes these data in a human accessible manner.

The process of graphical user interface design should be heavily influenced by principles of consistent and appealing graphical design [21], information visualization, and user-specific needs (see Rule 4) [22]. It is useful to employ a process of iterative design, in which feedback from potential users or experienced advisors about the interface is collected before, during, and after the development of the design [23,24]. Interfaces should immediately present the user with the most essential search and browse options and require the least amount of user actions (e.g., keyboard keystrokes, touchscreen taps) to reach the desired information in a speedy and responsive manner. Graphically appealing and color blind–friendly [25] color palettes such as Color Brewer (http://colorbrewer2.org/) are now standard and should be used when choosing a color scheme for both your web interface and data visualization. Interactive data visualization libraries, such as D3.js and BioJS [26,27], are useful sources of visualization methods to display all or selected subsets of large, complex, and heterogeneous biological data sets [28]. For example, jsPhyloSVG [29] draws phylogenetic trees and Cytoscape.js [30] draws networks. Often an artistically talented student can be recruited to help with this, but consider hiring a professional consultant on larger projects.

### Rule 6: Keep search simple and organized

Search options should help users precisely and quickly find what they are looking for. Most users are casual and rely on quick access to data via web-based search functions; thus, it is a good idea to design the default search functionality to meet a small set of the most frequent use cases. Minimally, a keyword search should be available that ideally provides a simple “Google-like” search term input form. It is likely that an advanced query system is both time consuming to implement and will be infrequently used; thus, addressing less frequent use cases can be decided based on resources available or could be left to advanced users to address themselves using available APIs. In all cases, it is useful to include a feature that loads an example query into the search system to help the user quickly figure out the correct input data types and format.

The organization of search results is important for enabling the user to quickly identify relevant results. First, search results should be grouped by the most important data type according to your database content. Second, if your query system accepts multiple simultaneous term searches (e.g., multiple genes in a gene database), group the results by search term. Third, sort the results by relevance, from more relevant to less relevant by following standard data mining approaches, such as term frequency–inverse document frequency (TF-IDF) [31]. These are usually available in standard text indexing systems, like Lucene. Fourth, add refinement or filtering options that enable interactive narrowing of the results, as implemented, for example, in the iRefWeb web interface for protein interaction data [32]. A well-developed refinement option is faceted search [33]. Fifth, show key information first, and then enable the user to ask for more, for instance, by grouping results into tabs or web page sections by data type. Sixth, summarize the results by showing the total number of results, including a breakdown by important categories.

### Rule 7: Give users data where they need it

Most users will likely find data access via a user-friendly, interactive website to be most convenient. However, users working with data in batch will find this to be inconvenient. Instead, they will want to download all the data in easy to process files. It is also useful to release at regular intervals to help users know when they should update and to archive previous data releases to help users reproduce published results based on older versions. Those accessing data from within software will want access to a web service API to program using. Use of your content can be amplified by copying to secondary databases, like GeneCards [34]. Each access channel should include well-written documentation and worked examples showing how to use the system.

### Rule 8: Support open science

Creating a database is a valuable intellectual contribution and a lot of work—you should feel proud to share it. Publish your data model or ontology in a journal and your source code in an open source venue, such as GitHub (http://www.github.com). Sharing source code enables others to contribute, fix the system if the original maintainer loses interest, provide reusable code components for other developers, and provide example code for training purposes. You may even make reusable software a goal of your project, such as with the Generic Model Organism Database (GMOD) for hosting model organism data [35]. Not only does the open access model provide great benefits to the community and is considered a best practice in the field [36], it is increasingly required by journals and funding agencies.

### Rule 9: Tell the world

Building your database and making it available online is unfortunately not enough to get people to use it—it needs to be actively promoted. First, publish an article describing your database in an appropriate journal. We recommend publishing in a journal that is likely to be read by the intended user community, though it may also be useful to publish in a journal devoted to database descriptions, such as the *NAR* database or web service issue, or *Database*: *The Journal of Biological Databases and Curation*. Second, index your website in popular search engines so that your database appears towards the top of the search results when searching related topics. This requires knowledge of search engine optimization techniques such as providing good and unique content, a stable website, and proper indexing of deep content [37]. Third, register your database in specialized online directories that list similar resources, such as *OMICtools* [10]. Fourth, promote your database in scientific conferences and meetings. Fifth, monitor online user groups, such as biostars.org, for potential users and let them know about your resource. Sixth, actively use social media to attract new users and keep them up to date with news about your resource [38]. Using social media can attract many users but requires sustained effort to be effective. If you cannot provide this effort over time, you shouldn’t create any social media content, as it will quickly go out of date and reflect badly on your resource (as we discovered in our GeneMANIA project). Finally, it is important to track usage to optimize database usability, promotion activities, and generally to measure how useful the database is to the community. Accurate tracking is difficult, especially if there are many distributed channels for data access, but web analytic tools, such as Google Analytics (https://analytics.google.com), and monitoring online mentions and citations can help. It is particularly useful to track examples where users have relied on the database to make new discoveries. This can be accomplished by scanning all papers that cite or mention your database and by personally discussing the utility of your database with users at conferences.

### Rule 10: Maintain, update, or retire

Maintaining a database is important for science reproducibility, and many research projects may depend on it being available far into the future. Many journals require a minimum period of at least two years of database maintenance for publication, and funding agencies require sustainable data sharing practices for continued support. Finally, failing to maintain a needed resource that you created can negatively affect your reputation and lead to reduced ability to publish similar work in the future.

Databases aiming for long life are often maintained by major institutions or consortia who employ dedicated staff to maintain content, software, and user support. On the other hand, the majority of databases are developed in small labs and mainly through personnel, such as students, with non-permanent positions [5]. In these cases, the database may become unsustainable because of lack of funding or career changes by interested individuals, which can eventually lead to its disappearance [39].

Fortunately, there are many technologies to help with sustainability on the technology side. First, use professionally managed hosting resources (e.g., cloud, institutional), instead of setting up your own web server. Second, if you must run your own server, use virtualization technology, like Docker, to ease system administration and backup tasks. If the server hardware fails, you can bring the system back quickly on another machine. Third, make your database available for download as a set of files or as an all-in-one virtual machine so that the system can be mirrored by others in case the central server is not available. Virtual machines can be contributed to free online repositories (e.g., https://hub.docker.com/). Fourth, regularly backup (and test restoration) your contents to avoid loss of information due to unfortunate technical problems—this is taken care of if you’re on the cloud. Fifth, make your database URL institution-independent (e.g., use.org or.net) to avoid breaking URL changes. Sixth, automatically monitor your system availability and test its main functions (e.g., with https://sensuapp.org/). If your site fails, the system will email you so you can fix it quickly. Seventh, provide means for users to report bugs and request features, such that your database can be improved. GitHub (https://github.com) provides a useful and free issue tracker and is automatically available if you host your source code there. Eighth, choose development technologies that are free and popular, as this will increase the chances that someone else will be available to fix or extend the system if needed. Ninth, if the database is outdated or can no longer be maintained, switch it off, but archive it publicly (e.g., on https://zenodo.org/ or a free virtual machine [VM] repository) so that others can resurrect it if needed [5].

Maintaining content is more challenging, and a significant effort is required to keep the contents of the database up to date, well documented, accurate, and comprehensive [15,16]. For meta-databases that draw content from other sites, content aggregation should be automated at regular intervals from the beginning of the project using tools like snakemake [40]. For high-quality curated databases, there is currently no replacement for the time consuming curation process; however, crowdsourcing is a promising research area that may already be able to help with certain tasks and, in the future, may greatly improve curation efficiency [41]. Sustainability of community-run databases is a hot discussion topic that we expect to generate new solutions for this challenge.

We hope that these ten rules provide a useful checklist and set of pointers to the literature for new database projects. Please visit our dynamic resource for recommended tools, technologies, and libraries at http://baderlab.org/TenRulesResources. Now get out there and collect and share data in a computable form!

## References

[1] Rigden DJ, Fernández-Suárez XM, Galperin MY. The 2016 database issue of Nucleic Acids Research and an updated molecular biology database collection. Nucleic Acids Res. Oxford University Press; 2016;44: D1–6. 10.1093/nar/gkv1356 26740669 PMC4702933

[2] Küntzer J, Eggle D, Klostermann S, Burtscher H. Human variation databases. Database (Oxford). 2010;2010: baq015. 20639550 10.1093/database/baq015 PMC2911800

[3] Soussi T. Locus-specific databases in cancer: what future in a post-genomic era? The TP53 LSDB paradigm. Hum Mutat. 2014;35: 643–53. 10.1002/humu.22518 24478183

[4] Galperin MY, Rigden DJ, Fernández-Suárez XM. The 2015 Nucleic Acids Research Database Issue and molecular biology database collection. Nucleic Acids Res. 2015;43: D1–5. 10.1093/nar/gku1241 25593347 PMC4383995

[5] Schultheiss SJ. Ten simple rules for providing a scientific Web resource. PLoS Comput Biol. 2011;7: e1001126 10.1371/journal.pcbi.1001126 21637800 PMC3102757

[6] Benson G. Editorial: Nucleic Acids Research annual Web Server Issue in 2015. Nucleic Acids Res. 2015;43: W1–2. 10.1093/nar/gkv581 26136473 PMC4489291

[7] Bader GD, Cary MP, Sander C. Pathguide: a pathway resource list. Nucleic Acids Res. 2006;34: D504–6. 10.1093/nar/gkj126 16381921 PMC1347488

[8] Ison J, Rapacki K, Ménager H, Kalaš M, Rydza E, Chmura P, et al Tools and data services registry: a community effort to document bioinformatics resources. Nucleic Acids Res. 2015;44: D38–47. 10.1093/nar/gkv1116 26538599 PMC4702812

[9] Brazas MD, Yim DS, Yamada JT, Ouellette BFF. The 2011 Bioinformatics Links Directory update: more resources, tools and databases and features to empower the bioinformatics community. Nucleic Acids Res. 2011;39: W3–7. 10.1093/nar/gkr514 21715385 PMC3125814

[10] Henry VJ, Bandrowski AE, Pepin A-S, Gonzalez BJ, Desfeux A. OMICtools: an informative directory for multi-omic data analysis. Database (Oxford). 2014;2014: bau069–. 25024350 10.1093/database/bau069 PMC4095679

[11] Bolser DM, Chibon P-Y, Palopoli N, Gong S, Jacob D, Del Angel VD, et al MetaBase—the wiki-database of biological databases. Nucleic Acids Res. 2012;40: D1250–4. 10.1093/nar/gkr1099 22139927 PMC3245051

[12] Benson DA, Clark K, Karsch-Mizrachi I, Lipman DJ, Ostell J, Sayers EW. GenBank. Nucleic Acids Res. 2015;43: D30–5. 10.1093/nar/gku1216 25414350 PMC4383990

[13] Boutet E, Lieberherr D, Tognolli M, Schneider M, Bansal P, Bridge AJ, et al UniProtKB/Swiss-Prot, the Manually Annotated Section of the UniProt KnowledgeBase: How to Use the Entry View. Methods Mol Biol. 2016;1374: 23–54. 10.1007/978-1-4939-3167-5_2 26519399

[14] Mitchell A, Chang H-Y, Daugherty L, Fraser M, Hunter S, Lopez R, et al The InterPro protein families database: the classification resource after 15 years. Nucleic Acids Res. 2015;43: D213–21. 10.1093/nar/gku1243 25428371 PMC4383996

[15] Holliday GL, Bairoch A, Bagos PG, Chatonnet A, Craik DJ, Finn RD, et al Key challenges for the creation and maintenance of specialist protein resources. Proteins. 2015;83: 1005–13. 10.1002/prot.24803 25820941 PMC4446195

[16] Babbitt PC, Bagos PG, Bairoch A, Bateman A, Chatonnet A, Chen MJ, et al Creating a specialist protein resource network: a meeting report for the protein bioinformatics and community resources retreat. Database (Oxford). 2015;2015: bav063. 26284514 10.1093/database/bav063 PMC4499208

[17] Hermjakob H, Montecchi-Palazzi L, Bader G, Wojcik J, Salwinski L, Ceol A, et al The HUPO PSI’s molecular interaction format—a community standard for the representation of protein interaction data. Nat Biotechnol. 2004;22: 177–83. 10.1038/nbt926 14755292

[18] Coordinators NR. Database resources of the National Center for Biotechnology Information. Nucleic Acids Res. 2016;44: D7–D19. 10.1093/nar/gkv1290 26615191 PMC4702911

[19] Gillenwater ZM. Stunning CSS3: A Project-based Guide to the Latest in CSS 1st ed. New Riders; 2010.

[20] Bolchini D, Finkelstein A, Perrone V, Nagl S. Better bioinformatics through usability analysis. Bioinformatics. 2008;25: 406–412. 10.1093/bioinformatics/btn633 19073592

[21] Marcus A. Graphic Design for Electronic Documents and User Interfaces. New York, NY, USA: ACM; 1992.

[22] Munzner T. Visualization Analysis and Design [Internet]. Florida, USA: CRC Press; 2014 Available: https://www.crcpress.com/Visualization-Analysis-and-Design/Munzner/p/book/9781466508910

[23] Pavelin K, Cham JA, de Matos P, Brooksbank C, Cameron G, Steinbeck C. Bioinformatics meets user-centred design: a perspective. PLoS Comput Biol. 2012;8: e1002554 10.1371/journal.pcbi.1002554 22807660 PMC3395592

[24] Bastien JMC. Usability testing: a review of some methodological and technical aspects of the method. Int J Med Inform. 2010;79: e18–23. 10.1016/j.ijmedinf.2008.12.004 19345139

[25] Wong B. Color blindness. Nat Methods. 2011;8: 441 Available: http://www.ncbi.nlm.nih.gov/pubmed/21774112 21774112 10.1038/nmeth.1618

[26] Bostock M, Ogievetsky V, Heer J. D^3^: Data-Driven Documents. IEEE Trans Vis Comput Graph. 2011;17: 2301–9. 10.1109/TVCG.2011.185 22034350

[27] Corpas M, Jimenez R, Carbon SJ, García A, Garcia L, Goldberg T, et al BioJS: an open source standard for biological visualisation—its status in 2014. F1000Research. 2014;3: 55 10.12688/f1000research.3-55.v1 25075290 PMC4103492

[28] Wang R, Perez-Riverol Y, Hermjakob H, Vizcaíno JA. Open source libraries and frameworks for biological data visualisation: A guide for developers. Proteomics. 2014; 10.1002/pmic.201400377 PMC4409855 25475079

[29] Smits SA, Ouverney CC. jsPhyloSVG: a javascript library for visualizing interactive and vector-based phylogenetic trees on the web. PLoS ONE. 2010;5: e12267 10.1371/journal.pone.0012267 20805892 PMC2923619

[30] Franz M, Lopes CT, Huck G, Dong Y, Sumer O, Bader GD. Cytoscape.js: a graph theory library for visualisation and analysis. Bioinformatics. 2015; btv557. 10.1093/bioinformatics/btv557 PMC4708103 26415722

[31] Azam N, Yao J. Comparison of term frequency and document frequency based feature selection metrics in text categorization. Expert Syst Appl. 2012;39: 4760–4768.

[32] Turner B, Razick S, Turinsky AL, Vlasblom J, Crowdy EK, Cho E, et al iRefWeb: interactive analysis of consolidated protein interaction data and their supporting evidence. Database (Oxford). 2010;2010: baq023. 20940177 10.1093/database/baq023 PMC2963317

[33] Tunkelang D. Faceted Search. Synth Lect Inf Concepts, Retrieval, Serv. Morgan & Claypool Publishers; 2009;1: 1–80.

[34] Safran M, Dalah I, Alexander J, Rosen N, Iny Stein T, Shmoish M, et al GeneCards Version 3: the human gene integrator. Database (Oxford). 2010;2010: baq020. 20689021 10.1093/database/baq020 PMC2938269

[35] O’Connor BD, Day A, Cain S, Arnaiz O, Sperling L, Stein LD. GMODWeb: a web framework for the Generic Model Organism Database. Genome Biol. 2008;9: R102 10.1186/gb-2008-9-6-r102 18570664 PMC2481422

[36] Stajich JE, Lapp H. Open source tools and toolkits for bioinformatics: significance, and where are we? Brief Bioinform. 2006;7: 287–96. 10.1093/bib/bbl026 16899494

[37] Inc. G. Search Engine Optimization Starter Guide. In: Google Inc. [Internet]. 2010 [cited 29 Jul 2015]. Available: http://static.googleusercontent.com/media/www.google.com/en//webmasters/docs/search-engine-optimization-starter-guide.pdf

[38] Bik HM, Dove ADM, Goldstein MC, Helm RR, MacPherson R, Martini K, et al Ten simple rules for effective online outreach. PLoS Comput Biol. Public Library of Science; 2015;11: e1003906 10.1371/journal.pcbi.1003906 25879439 PMC4399988

[39] Wren JD. URL decay in MEDLINE—a 4-year follow-up study. Bioinformatics. 2008;24: 1381–5. 10.1093/bioinformatics/btn127 18413326

[40] Köster J, Rahmann S. Snakemake—a scalable bioinformatics workflow engine. Bioinformatics. 2012;28: 2520–2. 10.1093/bioinformatics/bts480 22908215

[41] Khare R, Good BM, Leaman R, Su AI, Lu Z. Crowdsourcing in biomedicine: challenges and opportunities. Brief Bioinform. 2015; bbv021–. 10.1093/bib/bbv021 PMC4719068 25888696

